# The Impact of Hemoglobin Transfusion Thresholds in Moderate-to-Severe Blunt Traumatic Brain Injury on 6-Month Neurologic Outcomes: A Systematic Review and Meta-Analysis

**DOI:** 10.3390/neurolint18060119

**Published:** 2026-06-19

**Authors:** Faraz Behzadi, Thomas C. Varkey, Shan Rizvi, Zana Alattar, Chase Seiter, Sydni Martinez, Allison J. Tompeck, Khalid Alsherbini

**Affiliations:** 1 Department of Neurology, College of Medicine, University of Arizona, Phoenix, AZ 85006, USA; 2 Department of General Surgery, College of Medicine, University of Arizona, Phoenix, AZ 85006, USA; 3 College of Pharmacy, University of Arizona, Phoenix, AZ 85006, USA

**Keywords:** traumatic brain injury, hemoglobin threshold, long-term outcomes, packed red blood cell transfusion

## Abstract

Introduction: Moderate-to-severe TBI (msTBI) disproportionately affects younger populations with high mortality and severe morbidity amongst survivors. A higher hemoglobin level has been suggested to improve oxygen delivery to the injured brain, and recent randomized trials revealed that more liberal hemoglobin (Hgb) transfusion thresholds may improve 6-month neurologic functional outcomes measured by Glasgow Outcome Scale Extended (GOS-E). This article aims to perform a comprehensive meta-analysis of functional neurologic outcomes and early mortality in msTBI patients with liberal (8 g/dL) versus restrictive (7 g/dL) hemoglobin (Hgb) transfusion thresholds. Methods: The Medline, Embase, and Cochrane databases were searched for primary literature concerned with msTBI and early Hgb transfusion thresholds, from inception to October 2025. Risk of bias was assessed for all selected articles. With a common-effect model, we estimated the pooled odds ratio of the primary outcome (6-month unfavorable outcome defined as GOS-E ≤ 4 or 5) and the secondary outcome (30-day mortality) for liberal versus restrictive Hgb transfusion thresholds. Results: After reviewing 484 articles, 12 met the inclusion criteria with 8 reporting 6-month functional neurological outcomes and 10 that reported 30-day mortality. After a direct comparison of 5208 cumulative patients, those with more liberal transfusion thresholds had a statistically significant reduction in unfavorable outcomes at 6 months (OR = 0.67; 95% CI [0.58–0.77]; *p* < 0.0001) compared to those with restrictive thresholds. Liberal transfusion thresholds showed no significant effect on 30-day mortality with the direct comparison of 4589 cumulative patients (OR = 0.93, 95% CI [0.78–1.11]). Conclusions: Higher Hgb transfusion thresholds in patients presenting with msTBI can improve functional outcomes at 6 months with a lack of significant effects on 30-day mortality.

## 1. Introduction

Moderate-to-severe acute blunt traumatic brain injury (msTBI) affects younger (average 6th decade of life) and disproportionately (70%) male populations [1]. Severe TBI has high early mortality (up to 40%) and severe morbidity amongst survivors [2]. Extensive prior research has revealed age, initial neurologic exams via the Glasgow Coma Score (GCS), head imaging findings, and hypoxic injury as clinical risk factors of poor outcomes in msTBI [3,4,5]. Early intracranial pathology in msTBI patients with impaired autoregulation concomitantly along with hemodynamic shock and impaired cerebrovascular autoregulation can significantly reduce brain tissue perfusion and oxygenation, leading to hypoxic injury [6].

Treating severe anemia has been shown to significantly reduce mortality in critically ill patients via improved tissue oxygen delivery capacity [7]. However, the liberal (8 g/dL) hemoglobin (Hgb) threshold for the transfusion of packed red blood cells (pRBCs) is not indicated in patients with various critical illnesses, especially those in the medical intensive care unit (ICU) with multiple comorbidities to avoid heart failure exacerbation and acute respiratory distress syndrome [8,9]; recent evidence shows that otherwise healthy, younger msTBI patients may benefit from higher Hgb transfusion thresholds in the acute setting [10].

The Hgb threshold of patients with neurological injuries has been a subject of multiple trials [11]. Some trials suggested a possibility of harm with higher transfusion thresholds, but this effect was highly confounded by the use of erythropoietin in patients with closed head injuries [12]. However, recent randomized clinical trials (RCTs) including TRAIN, HEMOTION, and others have studied the long-term benefits of liberal (8, 9 or 10 g/dL) vs. restrictive (7 g/dL) Hgb transfusion thresholds in msTBI patients [11,13,14,15].

This study aims to perform a comprehensive meta-analysis and review of all RCTs and appropriately sized retrospective peer-reviewed studies that studied the functional neurologic outcomes of different Hgb transfusion thresholds in msTBI patients.

## 2. Methods

An institutional board review was not required for this study because this study does not involve any patients/subjects and only serves as a review of already peer-reviewed publications in the literature. No participants were involved in this study; therefore, no informed consent was required. This meta-analysis was registered in PROSPERO under registration ID 1376818. This meta-analysis was done in accordance of PRISMA guidelines (Table S1).

Medline, Embase, and the Cochrane Library were systematically searched from inception through October 2025. The search strategy used a combination of the following terms: *(TBI OR traumatic brain injury OR brain injury) AND (transfusion OR hemoglobin OR RBC OR Hgb OR blood products)*. These terms were restricted to the title and abstract fields. Eligible studies included randomized controlled trials, prospective comparative studies, observational cohort studies, case–control studies, and retrospective peer-reviewed investigations that evaluated functional Glasgow Outcome Scale Extended (GOS-E) or mortality outcomes at approximately 6 months in adult patients with msTBI, comparing different hemoglobin levels or red blood cell transfusion thresholds (i.e., with Hgb levels below this threshold, patient would have been transfused with pRBCs). GOS-E is a highly validated 8-point scoring system that has been a central tool for evaluation of functional neurologic outcomes in TBI patients [16].

Only studies published in English were included. Titles and abstracts were independently screened, followed by full-text review. Moderate-to-severe traumatic brain injury was defined as a Glasgow Coma Scale ≤ 12, a duration of post-traumatic amnesia greater than 24 h, or radiographic evidence of a skull fracture or intracranial hemorrhage. Studies were excluded if they were non-clinical (basic science or animal studies), pediatric cases (age < 18 years), case reports, or small case series (without a comparison group). Studies were also excluded if they included confounding intervention arms that prevented isolation of transfusion or hemoglobin threshold effects or if they exclusively enrolled patients with mild traumatic brain injury or penetrating brain injury.

All studies underwent appropriate bias assessment based on their nature. The Review of Bias (RoB-2) tool was applied to assess the risk of bias in randomized studies. Risk of Bias in Non-randomized Studies Intervention (ROBINS-I) was used for non-randomized prospective studies, and ROBINS-E (for Exposure) was used for retrospective studies. All appropriate (five) metrics of bias were reviewed by the authors (FB and TV) and were rated on a 3-grade scale of low, intermediate, and high.

All included studies were grouped based on their availability of outcome data for review: (1) 6-month functional outcomes assessed by the GOS-E score and (2) 30-day mortality. The number of total subjects in each arm or cohort of the study was reported as well as the overall average age and distribution of sex (if data available). The patient arms and cohorts were defined by the transfusion thresholds: restrictive (7 g/dL) vs. liberal (8 g/dL). The primary outcome of this study was unfavorable 6-month functional neurologic outcomes, which were defined as GOS-E ≤ 4 or 5 based on individual studies. The secondary outcome was 30-day mortality.

The distribution of primary and secondary outcomes was calculated in both arms of each included study. Then using R-studio (version 2023.06.1+524), a Mantel–Haenszel test was used to calculate the pooled odds ratio. Both common- and random-effects models were used, and given the significant heterogeneity amongst population sizes, a common-effect model was ultimately used.

## 3. Results

**Figure 1:**
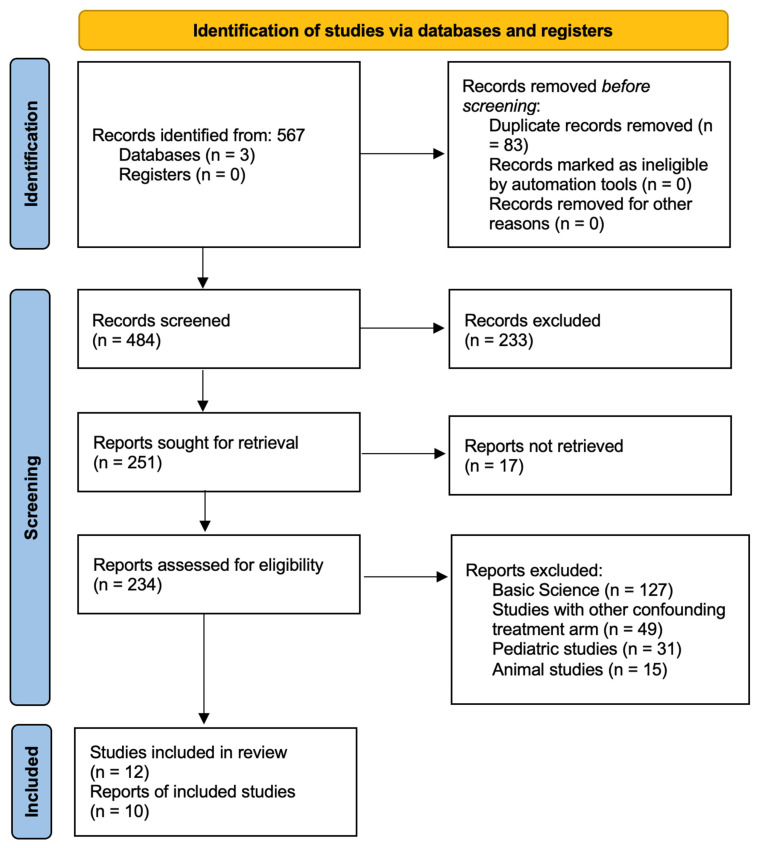
Flowchart for selecting the included and excluded peer-reviewed.

A total of 484 articles met the initial search inclusion criteria across all three databases. After reviewing the articles for exclusion criteria, 233 were irrelevant studies, 127 were basic science studies, 31 were pediatric studies, 15 were animal studies, and 49 had other confounding treatment arms. Amongst the remaining 29 articles, only 12 had accessible data for the purpose of this study; eight had 6-month GOS-E outcome data and 10 had 30-day mortality outcomes (Figure 1). Amongst the eight studies with 6-month GOS-E outcomes, five were randomized studies (three RCTs), two were non-randomized prospective studies, and one was a retrospective study; amongst the 10 studies with mortality outcomes, five were randomized studies (three RCTs), four were non-randomized prospective studies, and one was a retrospective study (Table 1). The various studies’ liberal transfusion thresholds are also included in Table 1.

The average age of the patients included in these 12 studies was 47 years with an approximate weighted standard deviation of 10. The ratio of females in these studies ranged from 13% to 46% but the majority of the studies were close to 20–30%. The year of publication of these studies was from 2008 to 2024, with the majority being from the 2010s. A detailed review of the literature did not reveal any major critical commentary on these 12 publications.

**Figure 2:**
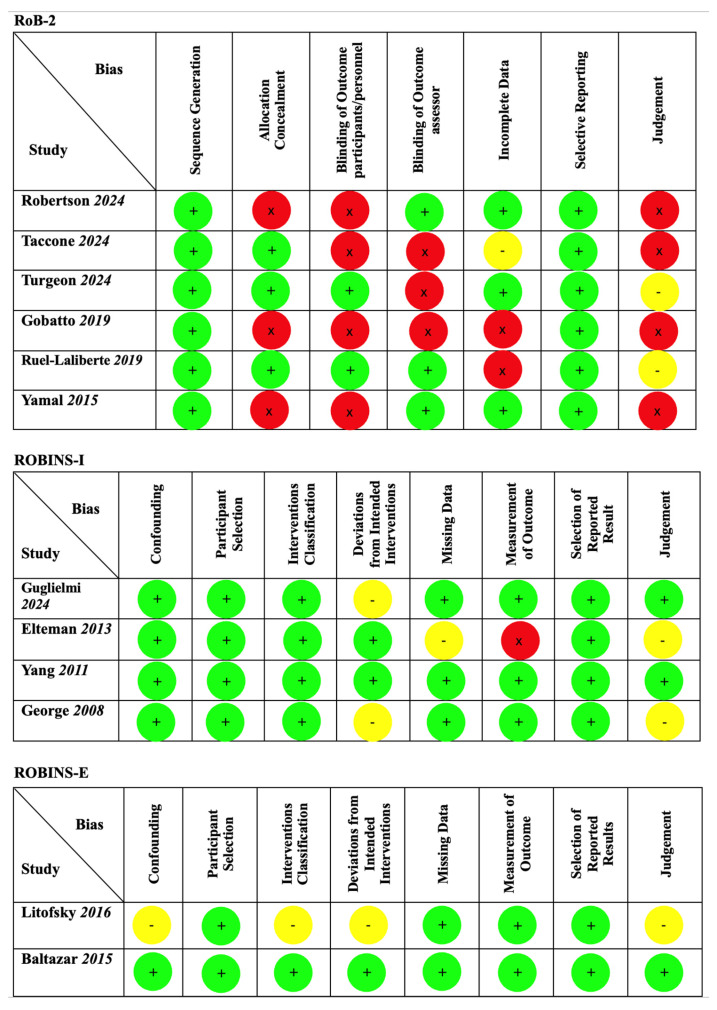
Risk-of-bias assessments: The Review of Bias (RoB-2) tool was applied to assess the risk of bias in randomized studies. Risk of Bias in Non-randomized Studies Intervention (ROBINS-I) was used for non-randomized prospective studies, and ROBINS-E (for Exposure) was used for retrospective studies. Green indicates a low risk of bias; yellow indicates an intermediate risk of bias, and red indicates a high risk of bias [11,12,13,17,18,19,20,21,22,23,24,25].

Amongst the eight studies with available 6-month GOS-E outcomes, the risk of bias revealed a generally low risk of randomization and confounders in all studies, a low risk of allocation concealment for most studies, and low risks of selective reporting, measurement bias, and missing data. However, detection bias and attrition biases were not as strong amongst the randomized but non-controlled studies (Figure 2).

**Figure 3:**
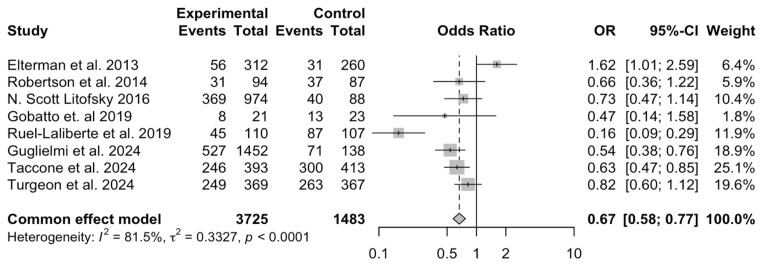
Forest plots comparing the unfavorable GOS-E rates of liberal and restrictive transfusion strategies in msTBI patients [11,12,13,17,18,19,20,23].

Amongst the eight studies with available data on 6-month GOS-E outcomes (a total of 5208 cumulative patients), 1483 patients were part of the restrictive transfusion threshold arm/cohort, and 3725 patients were part of the liberal transfusion threshold arm/cohort. The Mantel–Haenszel test with a common-effect model revealed an I^2^ heterogeneity value of 81.5%. The liberal transfusion threshold group was significantly less likely to experience unfavorable outcomes at 6 months (OR = 0.67; 95% CI [0.58–0.77]). A more detailed patient distribution of unfavorable outcomes is presented in Figure 3.

**Figure 4:**
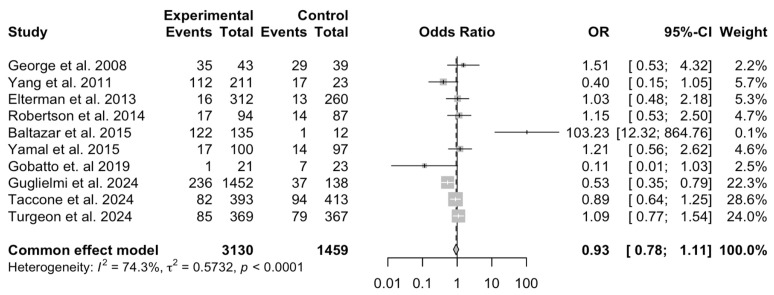
Forest plots comparing the mortality rates of liberal and restrictive transfusion strategies in msTBI patients [11,12,13,17,18,21,22,23,24,25].

Amongst the 10 studies with available data on 30-day mortality (total of 4589 cumulative patients), 1459 patients were part of the restrictive transfusion threshold arm/cohort, and 3130 patients were part of the liberal transfusion threshold arm/cohort. The Mantel-Haenszel with common effect model revealed no significant difference in 30-day mortality between the restrictive and liberal groups (OR = 0.93; 95% CI [0.78, 1.11]). A more detailed patient distribution of mortality outcomes is presented in figure format (Figure 4).

## 4. Discussion

The results of our meta-analysis (among the eight studies with available GOS-E data) demonstrate that in patients with traumatic brain injury, there is a significant reduction in unfavorable 6-month neurologic outcomes, as defined by GOS-E ≤ 4 or 5 at 6 months, with a liberalized packed red blood cell (pRBC) transfusion threshold (mainly Hgb 9 or 10 g/dL) as compared to a more restrictive threshold (7 g/dL). However, there were no significant difference in early 30-day mortality between these two groups.

### 4.1. 6-Month Neurologic Outcomes

Multiple retrospective studies included in our study, which were not included in those by Wang et al. and Machado et al., have demonstrated that the severity of anemia, both during the initial hospitalization and the hospital course, directly correlates with poor 6-month neurologic outcomes, suggesting that maintenance of appropriate Hgb levels is an important aspect of these patients’ medical management in the ICU [15,17,20]. Since the study by Wang et al. was published, there has also been new data which sways the results more towards those of liberal transfusion, as seen in the study by Guglielmi et al., who performed a secondary analysis of the CENTER-TBI data and found that hemoglobin increase during the hospital stay was independently associated with a decrease in the 6-month unfavorable neurological outcomes.

Recent RCTs studying the effects of restrictive (7 g/dL) versus liberal (8, 9, or 10 g/dL) Hgb transfusion thresholds have shown varying early and 6-month outcomes. While most have demonstrated a decrease in 6-month unfavorable neurological outcomes (defined by GOS-E ≤ 4 or 5) with liberal thresholds [13,18], they generally have not found significant differences in early outcomes, with some studies deeming liberal thresholds to be associated with higher incidences of adverse events [26].

### 4.2. The Impact on Early Mortality

According to our analysis, although higher transfusion thresholds did improve the early mortality rate in msTBI, the results of this meta-analysis did not reveal an increase in early mortality rates between higher or lower Hgb transfusion thresholds. Although the concern for transfusion complications such as acute respiratory distress syndrome (ARDS), heart failure exacerbation, and other transfusion syndromes remain a main concern in ICU patients, the most feared end result, early mortality, is not higher in the more liberal transfusion groups.

Other studies, conversely, have shown that lower Hgb levels in the hospital were not associated with increased short-term mortality [27]. One possible yet not complete explanation for this neutrality in early 30-day mortality is that early survival in msTBI patients is most linked to age, hemodynamic stability, chronic cardiovascular/pulmonary disease status, and non-neurologic medical complications that are often unrelated to less optimal oxygen delivery capacity [28,29]. This is as opposed to the more insidious neurologic deficit that usually reveals itself later during the 6-month evaluation of these patients. Some retrospective and comparative studies demonstrated an increase in early mortality when msTBI patients were transfused with pRBCs without a Hgb transfusion threshold or one above 10 g/dL [20,23,24,25]. It is important to note that transfusion of pRBCs is not free of risk; heart failure patients may experience a fatal fluid overloaded state, and rapid transfusion syndromes such as transfusion-associated circulatory overflow and transfusion-related acute lung injury, which lead to ARDS and death, in addition to reactions to transfusion and transfusion related lung injury TRALI, may occur [12,13,22,26]. Therefore, patient comorbidities should always be considered for transfusion thresholds.

### 4.3. Pathophysiology

Immediately after the mechanical brain injury from a blunt traumatic impact, msTBI patients often face multifactorial neurologic injury during the acute phase secondary to pathologic systemic responses such as inflammatory cerebral edema, increased intracranial pressure, reduced cerebral perfusion pressure, impaired cerebrovascular autoregulation, hypoxia, and hemodynamic instability, leading to improper tissue oxygenation and irreversible ischemic injury [30].

In msTBI, vasogenic edema due to the brain’s initial inflammatory response, cytotoxic edema due to cerebral contusion, and a change in available space due to skull fractures lead to elevated intracranial pressure (ICP) where cerebrovascular autoregulation is unable to maintain appropriate cerebral perfusion [31,32,33]. Furthermore, when patients are in shock, metabolic acidosis impairs autoregulation, causing an inappropriate increase in cerebral blood flow and a further ICP crisis [34]. Nonetheless, the impaired dynamics of cerebrovascular autoregulation are not readily treatable, and an easier focus of morbidity prevention may be putting more emphasis on modifiable metrics of brain tissue oxygenation such as control of oxygen delivery and anemia [35,36]. Recent studies evaluating the partial pressure of oxygen-targeted therapy found that it might potentially impact the outcome in a subset of patients with increased intracranial pressure, suggesting that for those with severely impaired autoregulation, the focus should be placed more on delivering normal perfusion pressure to the brain or just lowering the intracranial pressure [37,38]. Furthermore, an increase in Hgb not only ameliorates the oxygen carrying capabilities of the patients’ blood but also improves cerebrovascular microcirculatory modulation and carries antioxidant biochemical properties [39,40].

### 4.4. Hgb Transfusion Threshold

Post-traumatic anemia, marked by low Hgb levels, often occurs because of concomitant hemorrhagic blood loss or due to a critical illness in addition to significant blood draws in the ICU [41]. In a retrospective review of 1150 patients with TBI, 46% of the patients were anemic at some point during their first week of hospital stay. Among those patients, 76% received a blood transfusion [42]. Until recently, there has been no clear consensus on a threshold for packed red blood cell (pRBC) transfusion in msTBI patients beyond the global threshold of Hgb > 7 g/dL despite the understanding that these patients probably require higher tissue oxygen delivery capacity, as discussed previously. A recent meta-analysis did not conclude any mortality benefit of using higher Hgb transfusion thresholds [14]. However, the recent study did not comprehensively review the 6-month neurologic functional outcome data of all relevant trials, and it did not review any of the retrospective peer-reviewed databases relevant to the msTBI functional outcomes and Hgb transfusion thresholds. Therefore, a more unifying review of all encompassing studies is required because individual studies are underpowered, some results across studies are inconsistent, and no clear consensus exists in guidelines. It is important to note that a more liberal Hgb transfusion threshold does not serve as a treatment measure for msTBI; rather, it serves as an approach for the management of clinically plausible cases, but only as a means to prevent further complications.

### 4.5. Limitations

This study has the limitation of only including a total of 12 peer-reviewed articles, some of which were retrospective studies and some of which had a high risk of bias. The studies with a higher risk of bias had limited contribution to the overall statistical analysis. A major limitation was the presence of class imbalance between the number of patients with different transfusion thresholds, with liberal threshold groups in some retrospective studies including significantly more patients. Another limitation of this meta-analysis is that the results of individual studies, although not statistically significant, were not always in accordance with the combined statistical findings of the meta-analysis. The Hgb transfusion threshold (8, 9, or 10) and the unfavorable GOS-E definition (≤4 or 5) were also not the same in all the included studies. Although the result of this meta-analysis is statistically significant, this study is not in control of many possible confounders including but not limited to patients’ prior comorbidities, detailed mechanisms of injury, and other ongoing medical complications such as hemorrhagic shock, sepsis, and ARDS. Given the heterogeneity of the included studies, the meta-analysis did not evaluate for possible complications of higher Hgb transfusion thresholds other than the morality rates discussed. Lastly, the results of this study are only applicable to the general adult population and not necessarily the pediatric or geriatric population.

## 5. Conclusions

In conclusion, this comprehensive meta-analysis demonstrates that liberal hemoglobin transfusion thresholds (>8–10 g/dL) in msTBI patients are associated with significantly improved 6-month neurological outcomes, as defined by GOS-E ≤ 4 or 5, without an impact on early mortality. These findings suggest that optimizing cerebral oxygen delivery through higher hemoglobin targets may provide a neuroprotective benefit in otherwise young and previously healthy msTBI populations, supporting a more individualized approach to transfusion management in the acute phase. Although the results reinforce a potential benefit of a liberal transfusion strategy, this study’s heterogeneity and limited sample size highlight the need for larger, standardized randomized controlled trials to better define the optimal transfusion threshold.

## Figures and Tables

**Table 1:** A descriptive population review of the peer-reviewed studies included.

Study Author	PMID/Database	Year	6-Month GOS-E Data	30-Day Mortality Data	Total N	Mean Age (SD), year	Sex (F), %	Group Split	Restrictive N	Liberal N	GOS-E Cutoff	Restrictive w/Unfavorable, %	Liberal w/Unfavorable, %	*p*-Value GOS-E	Restrictive w/Mortality, %	Liberal w/Mortality, %	*p*-Value Mortality
Guglielmi et al. [17]	38877571	2024	Yes	Yes	1590	49 (19)	19	Hgb 8, 10	138	1452	<5	51	36	<0.001	27	16	0.01
Taccone et al. [13]	39382241	2024	Yes	Yes	850	51	46	Hgb 7, 9	413	393	<5	73	63	0.002	23	21	0.6
Turgeon et al. [11]	38869931	2024	Yes	Yes	742	48.9 (18)	27	Hgb 7, 10	367	369	<5	72	67	0.6	22	23	0.7
Gobatto et al. [18]	Cochrane	2019	Yes	Yes	44	35 (13)	9.1	Hgb 7, 9	23	21	<5	57	38	0.06	30	4.8	0.09
Ruel-Laliberte et al. [19]	30809776	2019	Yes	No	217	51 (21)	29	Hgb 7, 8	107	110	<5	81	41	0.35	N/A	N/A	N/A
Litofsky et al. [20]	26921698	2016	Yes	No	939	46.75 (24.6)	34	Hgb 7, 10	88	974	<5	45	38	<0.05	N/A	N/A	N/A
Baltazar et al. [21]	25789550	2015	No	Yes	147	54 (3.7)	Unknown	Hgb 10	12	135	N/A	N/A	N/A	N/A	8.3	90	0.02
Yamal et al. [22]	25566694	2015	No	Yes	197	Unknown	Unknown	Hgb 7, 10	97	100	N/A	N/A	N/A	N/A	14	17	0.7
Robertson et al. [12]	25058216	2014	Yes	Yes	200	Unknown	13	Hgb 7, 10	87	94	<4	43	33	0.28	16	18	0.8
Elterman et al. [23]	23778432	2013	Yes	Yes	1158	39.4	24	Hgb 7, 10	260	312	<4	12	18	0.41	5.0	5.1	0.4
Yang et al. [24]	21427611	2011	No	Yes	234	52 (18)	Unknown	Hgb 10	23	211	N/A	N/A	N/A	N/A	74	53	0.3
George et al. [25]	18273711	2008	No	Yes	82	53 (21)	33	Hgb 8, 10	39	43	N/A	N/A	N/A	N/A	74	81	0.8

## Data Availability

The original contributions presented in this study are included in the article/Supplementary Material. Further inquiries can be directed to the corresponding authors.

## Supplementary Materials

The following supporting information can be downloaded at https://www.mdpi.com/article/10.3390/neurolint18060119/s1, Table S1: PRISMA 2020 checklist.
